# Pathogenic potential of Shiga toxin-producing *Escherichia coli* strains of caprine origin: virulence genes, Shiga toxin subtypes, phylogenetic background and clonal relatedness

**DOI:** 10.1186/s12917-018-1407-2

**Published:** 2018-03-16

**Authors:** Maziar Jajarmi, Mahdi Askari Badouei, Abbas Ali Imani Fooladi, Reza Ghanbarpour, Ali Ahmadi

**Affiliations:** 1 Applied Microbiology Research Center, Systems Biology and Poisonings Institute, Baqiyatallah University of Medical Sciences, Tehran, Iran; 2 Department of Pathobiology, Faculty of Veterinary Medicine, Ferdowsi University of Mashhad, Mashhad, Iran; 3 Molecular Microbiology Research Group, Shahid Bahonar University of Kerman, Kerman, Iran; 4 Molecular Biology Research Center, Systems Biology and Poisonings Institute, Baqiyatallah University of Medical Sciences, Tehran, Iran

**Keywords:** Goats, phylogeny, serogroup, Shiga-toxin producing *Escherichia coli*, subtype

## Abstract

**Background:**

All over the world, Shiga toxin-producing *Escherichia coli* (STEC) are considered as important zoonotic pathogens. Eight serogroups have the greatest role in the outbreaks and diseases caused by STEC which include O26, O45, O103, O111, O113, O121, O145 and O157. Ruminants, especially cattle are the main reservoirs but the role of small ruminants in the epidemiology of human infections has not been thoroughly assessed in many countries. The objective of this research was to investigate the pathogenic potential of the STEC strains isolated from slaughtered goats. In this study, a total of 57 STEC strains were recovered from 450 goats and characterized by subtyping of *stx* genes, O-serogrouping, phylo-typing and DNA fingerprinting.

**Results:**

Amongst 57 STEC strains isolated from goats, the prevalence of *stx1* was significantly more than *stx2* (98.2% vs. 24.5%; *P* ≤ 0.05), and 22.8% of strains harbored both *stx1* and *stx2* genes. Three (5.2%) isolates were characterized as EHEC, which carried both *eae* and *stx* genes. A total of five *stx*-subtypes were recognized namely: *stx1c* (94.7%), *stx1a* (53.7%), *stx2d* (21%), *stx2c* (17.5%), and *stx2a* (15.7%). In some parts of the world, these subtypes have been reported in relation with severe human infections. The *stx* subtypes predominantly occurred in four combinations, including *stx1a/stx1c* (35%), *stx1c* (31.5%), *stx1c/stx2a/stx2c/stx2d* (5.2%) and *stx1c/stx2c/stx2d* (%5.2%). In serogrouping, the majority of STECs from goats did not belong to the top 8 serogroups but two strains belonged to O113, which has been recognized as an important pathogenic STEC in Australia. Interestingly, none of *stx*^*+*^*eae*^*+*^ isolates belonged to the tested serogroups. In phylo-typing the isolates mostly belonged to phylo-group B1 (82.4%), followed by phylo-group A (12.3%). STEC strains showed a substantial diversity in DNA fingerprinting; there were 24 unique ERIC-types (with a ≥95% similarity) among the isolates.

**Conclusions:**

Despite the fact that the top 8 STEC serogroups were uncommon in caprine strains, the presence of highly pathogenic *stx* subtypes indicates that small ruminants and their products can be considered as an overlooked public health risk for humans, especially in developing countries which consume traditional products.

## Background

Shiga toxin-producing *Escherichia coli* (STEC) are among the most challenging microorganisms of public health concern. This is because they are known as foodborne pathogens and are responsible for the sporadic and epidemic incidence of hemorrhagic colitis (HC) and hemolytic uremic syndrome (HUS), worldwide [1]. STECs are found in humans and a wide range of warm blooded animals, but ruminants have been proven as the main source of food contamination and the major reservoirs for human infections [2].

The pathogenic potential of STEC strains can be attributed to various factors, such as virulence genes and Shiga toxin subtypes [3]. Also, some characteristics like serotypes and phylo-types can be regarded as indicators of pathogenic STECs. Overall, there are two primary Shiga toxins (Stx1 and Stx2), and each of them is divided into different subtypes [4, 5]. At present, some studies have reported the relationship between some subtypes with HC and HUS infections e.g. Stx2a, Stx2c and Stx2d [6, 7].

Based on the role of diverse serotypes in human outbreaks, STECs can be categorized into O157 and non-O157 serogroups [8]. Recently, researchers in Europe and the United States have revealed that non-O157 serogroups such as O26, O45, O103, O111, O113, O121 and O145 are responsible for approximately one-thirds of STEC diseases in parallel with O157, collectively referred to as big eight O-serogroups [9, 10]. In contrast to developed countries, the distribution, and role of STEC serotypes are unknown in developing countries, especially in the Middle East region.

Numerous studies have been conducted on the frequency of STECs in ruminants [11, 12]; recently, comprehensive studies have been conducted on cattle which introduced them as very important reservoirs of non-O157 virulent STECs with potential pathogenicity for humans [13, 14]. Importantly, in 2015, we described the probable association of STEC from sheep and cases of uncomplicated diarrhea in children [15], but the overall public health risk associated with small ruminants STEC is unclear; some important traits like the Stx-subtypes are yet to be studied. In developing countries, meat and dairy products of small ruminants are abundantly produced and consumed in a traditional manner, which might have an impact on the epidemiology of foodborne diseases like STEC associated infections. Therefore, this project intends to investigate the pathogenic potential of Shiga toxin-producing *E. coli* strains of caprine origin through the screening of major virulence genes, Shiga toxin subtypes, big eight O-serogroups, phylogenetic background and clonal relatedness for the first time in Iran.

## Methods

### Collection of STEC isolates by screening of their primary virulence genes *(stx1*, *stx2* and *eae)*

A bacterial collection consisting of 57 caprine STEC strains was chosen for this study. Theses strains were collected during several steps including: (i) sampling from feces of 250 goats and of 200 goat carcasses by sterile swabs in slaughterhouses in Iran (Kerman, Khorasan, Fars, Sistan and Baluchestan) during 2013-2016 (fecal samples were prepared using rectal swabs and for sampling from carcasses, two sterile swabs were rubbed on each internal and external areas of carcasses), (ii) transferring to laboratory within 24 hours in Cary-Blair (Oxoid, United Kingdom) transport medium [15], (iii) cultivation on MacConkey agar (Merck, Germany) at 37°C for 24 h and isolation of suspected *E. coli* strains [16], (iv) confirmation of the isolates using biochemical tests including TSI (Triple Sugar Iron Agar), and IMViC (indole, methyl red, Voges-Proskauer, citrate) [16], (v) DNA extraction of *E. coli* isolates [17], (vi) molecular detection of STEC strains on the basis of main genetic markers (*stx1, stx2 and eae* genes) using a touchdown multiplex-PCR procedure explained by Paton and Paton (2002) [18], and (vii) storage of the 57 STEC (*stx*-positive) isolates in Luria Bertani (LB) broth (Merck, Germany) containing 20% glycerol which finally were stored at -80°C for next steps.

### Subtyping of *stx* genes

The glycerolated STEC strains were refreshed in Brain Heart Infusion (BHI) broth, (Merck, Germany) then plated on MacConkey agar and finally a single-colony was selected from each plate for DNA extraction. After DNA extraction via NaOH method [17], all *stx*-positive strains were subtyped via an accredited molecular technique described by Scheutz *et al.* (2012) [5] which includes a multiplex-PCR to find *stx1a*, *stx1c* and *stx1d* subtypes; and also seven simplex PCR to detect *stx2a*, *stx2b*, *stx2c*, *stx2d*, *stx2e*, *stx2f* and *stx2g* subtypes [14]. We performed further PCRs using a 66°C annealing temperature for elimination of false-positive bands.

### Molecular screening of top eight STEC serogroups

We evaluated attendance of eight important and human pathogenic serogroups (O26, O45, O103, O111, O113, O121, O145 and O157) among all *stx*-positive strains using a multiplex-PCR; this method has been developed by DebRoy et al. (2011) [19]. No other serotyping (especially by agglutination tests) was performed.

### Phylo-grouping of STEC strains

For identification of phylo-groups (A, B1, B2, C, D, E, F), this project employed a procedure determined by Clermont et al (2013) [20]; first, a quadruplex PCR was used to detect four sequences called *arpA* (400 bp), *chuA* (288 bp), *yjaA* (211 bp) and TspE4.C2 (152 bp). Then, complementary PCRs were carried out on the strains which were not classified into a particular phylo-group [20, 21].

### Clonal relatedness of STEC strains

All 57 STEC strains were selected to fingerprinting via ERIC-PCR (enterobacterial repetitive intergenic consensus sequences - polymerase chain reaction) technique for better understanding the clonal relationship among the *stx*-positive strains. This method was carried out using a pair of primers called ERIC1 and ERIC2 as presented by Versalovic et al. [22]. Initially, the bacterial strains were cultured in LB broth for an overnight at 37°C, DNA was extracted by a commercial kit (Cinnagen, Iran), ERIC-PCR was done, and finally electrophoresis (75 V) was conducted on 2% agarose gel for 3 h [15].

For evaluation of ERIC-PCR results, the pictures of the bands were recorded by Gel Doc 1000 imaging system (Vilber Lourmat, France). The banding patterns of images were calibrated and analyzed using a software named 1D Pro (Totallab, United Kingdom) and the similarity of ERIC-types was indicated by the drawing of a phylogenetic tree using the unweighted pair-group with mathematic average (UPGMA) clustering method; ≥95% Dice similarity cut-off were considered to identify the same clonal isolates, and 65% Dice similarity level was considered to comparative study of the STECs, in view of genotype and source.

### Statistical analysis

All data about the presence or absence of variables were entered into Excel (Microsoft 2016) and SPSS (SPSS 24; IBM) programs as binomial information for descriptive statistical analysis including calculation of prevalence percentages and confidence intervals. Then the pair-wise comparison of frequencies was done in a non-parametric binomial analysis; confidence level and *P* value were 95% and ≤0.05, respectively.

## Results

### Prevalence and combination patterns of *stx1*, *stx2* and *eae* genes in STEC strains

STEC strains were found in 14 meat and 43 fecal samples. So, the percentages reported in the present study have been mostly calculated on the basis of 57 *stx*-positive strains as the sample size. Overall, 43 (75.4%) isolates possessed only *stx1*, 13 (22.8%) strains were positive for both *stx1* and *stx2*, and only one (1.7%) isolate had just *stx2* gene (Table 1). So, 98.2% of our STEC strains were *stx1*-positive and it is notable that *stx1* was significantly (*P* < 0.05) more prevalent than *stx2* (24.5%). The *eae* gene was found in 3 (5.2%) isolates, introducing them as EHEC pathotype. Totally, Four diverse virulence profiles were determined which include: *stx1* (40 isolates), *stx1*/*stx2* (13 isolates), *stx1/eae* (3 isolates) and *stx2* (1 isolate); there was no significant difference between feces and meat about the prevalence of the virulence gene profiles (Table 2).

**Table 1:** Distribution of *stx* subtypes, *eae* gene, serogroups, phylogenetic groups and ERIC-types in 57 STEC isolates from goat

No. of isolates	*stx* subtypes and virulence genes							Pathotype (No. Source)	Phylo-group (No. Source)	ERIC-types (No. Source) with 65% Dice similarity
	*stx1a*	*stx1c*	*stx2a*	*stx2b*	*stx2c*	*stx2d*	*eae*			
20	+	+						STEC (16^F^, 4^M^)	B1(16^F^, 2^M^), A(1^M^), U(1^M^)	II(1^M^), IV(6^F^), V(2^F^, 1^M^), VI(2^F^), VIII(4^F^, 1^M^), IX(1^M^), X(2^F^)
18		+						STEC (13^F^, 5^M^)	B1(9^F^, 4^M^), A(4^F^, 1^M^)	I(1^M^), II(1^M^), IV(5^F^, 1^M^), VI(3^F^), VIII(2^F^, 1^M^), X(3^F^, 1^M^)
3		+			+	+		STEC (3^F^)	B1(3^F^)	VII(1^F^), VIII(1^F^), X(1^F^)
3		+	+		+	+		STEC (2^F^, 1^F^)	B1(2^F^), U(1^F^)	IV(1^F^), VI(1^F^), X(1^F^)
2	+	+	+			+		STEC (2^M^)	B1(2^M^)	V(1^M^), VIII(1^M^)
2	+							STEC (2^F^)	B1(2^F^)	II(1^F^), III(1^F^)
2		+					+	EHEC (1^F^, 1^M^)	B1(1^F^), U(1^M^)	IV(1^F^), VIII(1^M^)
1	+	+					+	EHEC (1^F^)	B1(1^F^)	IV(1^F^)
1		+				+		STEC (1^M^)	B1(1^M^)	VII(1^M^)
1	+	+			+	+		STEC (1^F^)	B1(1^F^)	VIII(1^F^)
1	+	+	+		+			STEC (1^M^)	B1(1^M^)	IX(1^M^)
1	+	+	+		+	+		STEC(1^F^)	B1(1^F^)	VIII(1^F^)
1 ^a^	+	+	+		+	+		STEC (1^F^)	A(1^F^)	X(1^F^)
1 ^a^			+	+				STEC (1^F^)	B1(1^F^)	IV(1^F^)
Total Source (43^F^, 14^M^)	29	54	9	1	10	12	3	STEC (41^F^, 13^M^), EHEC (2^F^, 1^M^)	B1(37^F^, 10^M^), A(5^F^, 2^M^), U(1^F^, 2^M^)	I(1^M^), II(1^F^, 2^M^), III(1^F^), IV(15^F^, 1^M^), V(2^F^, 2^M^), VI(6^F^), VII(1^M^, 1^F^), VIII(9^F^, 4^M^), IX(2^M^), X(8^F^, 1^M^)

*Abbreviations*: *EHEC* Enterohemorrhagic *Escherichia coli, STEC* Shiga toxin-producing *Escherichia coli*, *U* Unknown, *F* feces, *M* Meat

^a^ This isolate belonged to O113 serogroup

**Table 2:** Prevalence percentage of virulence gene profiles and phylo-groups among the STEC isolates from feces and meat

	Virulence gene profiles (No. of isolates)				Phylo-group (No. of isolates)		
Source	*stx1*(40)	*stx1*/*stx2*(13)	*stx1/eae* (3)	*stx2*(1)	A (7)	B1 (47)	U (3)
Feces (43)	72.1% (31)	20.9% (9)	4.7% (2)	2.3% (1)	11.6% (5)	86.1 (37)	2.3% (1)
Meat (14)	64.3% (9)	28.6% (4)	7.1% (1)	0 (0)	14.3% (2)	71.4% (10)	14.3% (2)

### Subtypes of *stx1* and *stx2* genes

Among 56 *stx1*-positive isolates, only two subtypes were found that includes: *stx1c* in 54 (94.7%) and *stx1a* in 29 (50.8%) strains. Also, we recognized five various *stx2* subtypes among fourteen strains that includes: *stx2d* (21.5%), *stx2c* (17.5%), *stx2a* (15.7%) and *stx2b* (1.7%). Besides, 11 profiles of *stx* subtypes were detected which *stx1a*/*stx1c* (21 strains), *stx1c* (20 strains), *stx1c*/*stx2a*/*stx2c*/*stx2d* (3 strains), and *stx1c*/*stx2c*/*stx2d* (3 strains) were the most frequent ones (Table 1).

### Detected O-serogroups and phylo-types

O-serogrouping showed that all STECs did not belong to O157. Two (3.5%) strains were found to be O113 and none of the other studied serogroups were identified in 55 remaining isolates (Table 1).

In phylo-grouping, results illustrated that majority of caprine STECs (82.4%) are significantly (*p* ≤ 0.05) associated with the B1 phylogenetic group. Genetic markers of phylo-group A were obtained from 12.3% of strains (Table 1), while phylo-type of 3 isolates (5.3%) were unknown. There was no significant difference between feces and meat about the prevalence of phylo-types in STEC isolates (Table 2).

### Clonal relatedness among STEC strains

In this research, *E. coli* isolates with a ≥95% Dice similarity cut-off were considered as the same clonal isolates. Therefore, 24 unique ERIC-types were specified amongst the 57 *stx*-positive isolates.

**Fig. 1:**
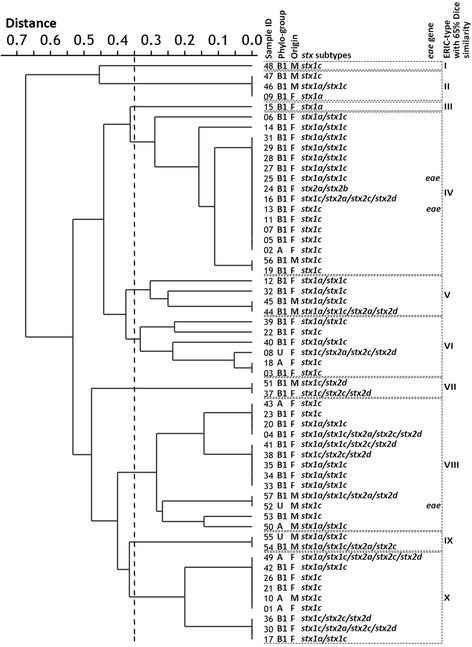
Dendrogram describing the relatedness of STEC strains obtained from the goats. The cut off is in 65% Dice similarity and the data of each isolates including phylo-group, origin of samples, primary virulence genes and *stx*-subtypes have mentioned after sample ID

Also, 65% similarity level was considered to compare the STECs in view of genotype and source. In this similarity cut-off, ten clusters (I to X) were defined (Fig. 1); 40 *stx1*^+^*stx2*^-^*eae*^-^isolates and 19 *stx1*^+^*stx2*^+^*eae*^-^ isolates were found in 9 and 8 ERIC-types, respectively. Two of the three *eae*^+^ strains had the same ERIC-type (IV). It is notable that in 6 ERIC-types, both feces and meat sources were observed.

## Discussion

In the present study, all goats had been sent to slaughterhouses for the production of raw meat. Also, most of the goats belonged to traditional farms where the goats have been exposed to cattle and sheep. These animals were in contact together at slaughterhouse in same holding pens. Accordingly, the molecular characterization of isolated STECs can clarify the role of healthy goats in the dissemination of this pathogen and pathogenic potential of caprine STECs.

In this study, frequency of *stx1* was extremely high and considerably more than *stx2*, in agreement with the researches on goats from 2003 till today. The gene *stx1* is often identified from diarrheic or asymptomatic cases, but *stx2* is mostly detected in HUS patients [23, 24]. Recently, *stx1*^*+*^*stx2*^*-*^*eae*^*-*^ strains have been reported from diarrheic children and sheep in Iran [15]. The simultaneous presence of *stx1* and *stx2* genes, which were found in about one-fifth of caprine isolates in this study, may increase the pathogenicity of such strains [25–27]. Also, the attendance of the *eae* gene in STEC strains resulted to the formation of a highly virulent sub-pathotype, EHEC, which was observed in three strains in the present survey; two EHEC isolates were in same ERIC-types. In this study, *eae* gene was found just in *stx1*^*+*^*stx2*^*-*^ isolates. This finding is in agreement with some previous studies about the feces and meat of goats [11, 28].

In the present survey, strains from goats were mostly negative for *eae*, which is similar to several previous researches in Europe and Asia. Hypothetically, these *eae*-negative strains may receive the genes encoding non-intimin adhesions via pathways of horizontal gene transfer [29]. The high percentage of our LEE-negative STEC corroborates the results of previous studies on all age groups of ruminants in Germany [30] and India [31]. It is noteworthy that some reports have shown that *eae*-negative STECs can also cause HC and HUS in humans, especially adult patients [32, 33].

Shiga toxin subtyping can improve understanding of the pathogenicity and epidemiology of STEC. In agreement with other studies on small ruminants and their products from 2008 to 2016 [27, 34–36], it was observed that *stx1c*, *stx1a*, *stx2d* and *stx2c* have approximately high prevalence. All *stx2*^+^ strains harbored at least one of the important subtypes including *stx2a*, *stx2c* or *stx2d*. These subtypes have been recognized in relation to severe STEC infections in humans [26]. This study detected the *stx2a* subtype in three-quarters of the *stx2-*containing isolates; this is comparable with the results reported from other hosts like cattle and wild ruminants [37]. This means that *stx2a* is not host specific and frequently occurs in different STEC reservoirs. Some strains simultaneously carried two or three different subtypes, such as *stx1a*/*stx1c, stx2a/stx2c/stx2d, stx2a/stx2c* and *stx2c/stx2d*. We recently encountered this phenomenon in a similar research on cattle [14], as it has been reported from humans [38] and pigs [39].

Significantly, all our STECs belonged to non-O157 serogroups; over the past 12 years, most studies have indicated the low prevalence of O157 in goats, except a report showing 36% frequency in goat meat [11]. In the present study, two STEC strains were classified as O113 that has contributed to the development of HUS and HC in Australia [40]. Some studies have repeatedly found several less common serotypes in goats such as O5, O126, and O146 that justify the lack or low prevalence of the top eight serogroups among STECs in this study [1, 27, 35]. In our previous research on cattle, three serogroups were detected; O113 (20%), O26 (12%) and O111 (10%) [14]. It can be concluded that ruminants are mainly the source of non-O157 STEC strains in our region and O113 could be considered as one of the most prevalent serogroups which may have the potential to cause infections like previous studies [41].

Analyses of the virulence genes, O-serogroups, ERIC-types and phylogenetic background data, in relation to each other, showed that our O113 strains contain important *stx2* subtypes including *stx2a, stx2c* and *stx2d*; this finding is similar to the reports from HUS cases, caused by *stx2*^+^/O113 strains [42] and healthy cattle [14] in some parts of the world. These strains were of two different phylogenetic and ERIC-types, showing their heterogeneity. Most members of phylo-group A just carried *stx1c*, excluding a clone which carried the combination of *stx* genes (*stx1a*/*stx1c*/*stx2a*/*stx2c*/*stx2d*); three of six strains belonging to phylo-group A were in the same ERIC-type (X) with 65% Dice similarity and the rest of them were distributed among three different ERIC-types (Fig. 1). The members of phylo-group B1 possessed a variety of virulence profiles without any significant clonal relationship. STECs of this study were found in ten (≥65% Dice similarity) to twenty four (≥95% Dice similarity) ERIC-types indicating their high genetic diversity (Fig. 1), which is similar to a study conducted in Turkey [43].

Generally, there was no significant difference between the characteristics of meat STECs with those obtained from feces which highlights the transferability of this pathogen via meat contaminated with feces. Molecularly, *stx*-carrying bacteriophages disseminate the *stx* genes and made the new STEC strains [44]. One-fourth of the STEC strains were isolated from meat; most of them belonged to phylo-group B1 and were categorized into 8 ERIC-types. In this study ERIC relatedness of *stx1c*^+^ strains originated from meat and feces was observed in three ERIC-types (IV, VIII and X). This issue was observed about *stx1a*^+^*stx1c*^+^ strains in one ERIC-type, too.

## Conclusions

This study showed caprine STECs may have the potential to cause human infections, suggesting the study of more virulence factors. Therefore, because of the very important position of goats in the production of meat and milk in developing countries, transmission to people may occur and cause mild to severe diseases. Since the big eight serogroups were not prevalent in goats compared to cattle, this host must be searched for other serogroups. Nevertheless, based on the findings of the present study, it was suggested that the O113 should be tested for LEE-negative STEC strains recovered from suspected human infections. The presence of highly pathogenic *stx* subtypes in the present study revealed the public health risk of caprine STEC for the first time in Iran.

## Abbreviations

*arpA*: Ankyrin-like regulatory protein A gene
BHI: Brain Heart Infusion
*chuA*: *E. coli* heme-utilization gene A gene
*eae*: *Escherichia coli* attaching and effacing gene
EHEC: Enterohemorrhagic *Escherichia coli* strains
ERIC: Enterobacterial repetitive intergenic consensus
HC: Hemorrhagic colitis
HUS: Hemolytic Uremic Syndrome
LB: Luria Bertani
LEE: Locus for enterocyte effacement
NaOH: Sodium Hydroxide
PCR: Polymerase Chain Reaction
SPSS: Statistical Package for the Social Sciences
STEC: Shiga toxin-producing *Escherichia coli*
Stx: Shiga toxin
TspE4.C2: an anonymous DNA fragment in *E. coli*
UPGMA: Unweighted Pair Group Method with Arithmetic Mean
*yjaA*: *E. coli* K12 gene
